# Short-term refractive and corneal astigmatism after canal-based microinvasive glaucoma surgery

**DOI:** 10.1371/journal.pone.0340377

**Published:** 2026-01-08

**Authors:** Colya N. Englisch, Karl T. Boden, André Messias, Philipp K. Roberts, André M. Trouvain, Peter Szurman, Achim Langenbucher, Philip Wakili

**Affiliations:** 1 Eye Clinic Sulzbach, Knappschaft Hospitals Saar, Sulzbach/Saar, Germany; 2 Department of Experimental Ophthalmology, Saarland University, Homburg/Saar, Germany; 3 Klaus Heimann Eye Research Institute (KHERI), Sulzbach/Saar, Germany; Alexandria University Faculty of Medicine, EGYPT

## Abstract

**Purpose:**

To investigate changes in refractive and corneal astigmatism after Hydrus Microstent (Alcon Inc., Geneva, Switzerland) implantation during microinvasive glaucoma surgery.

**Setting:**

One German medical center.

**Design:**

Retrospective, non-randomized, observational cohort study.

**Methods:**

Subjective refraction was obtained mandatorily and keratometry (Pentacam, OCULUS Optikgeräte GmbH, Wetzlar, Germany) optionally preoperatively and after 3 months (D90). Refractive astigmatism was analyzed using three-dimensional (3D) power vectors, including the spherical equivalent, *M*, the Jackson cross-cylinder projections to the 0° and 90° meridian (*J*_0_) and to the 45° and 135° meridian (*J*_45_), and the blurring power vector *B*. Corneal anterior and posterior surface astigmatism was investigated using the vectorial parameters *J*_0_, *J*_45_, and *B*. A 3D power vector time-course analysis of refractive astigmatism was performed using multivariate pairwise testing, and linear mixed-effects models, accounting for patient ID as a random effect, were used for the remaining analyses.

**Results:**

A total of 90 ocular hypertension/glaucoma eyes were included. Sixty eyes were implanted with a Hydrus Microstent and phacoemulsified, and 30 eyes underwent implantation as a stand-alone procedure. Univariate analysis of the refractive cylinder showed no variation over time in any cohort (Phacoemulsification: C_D0_ = –1.3 ± 0.9 D vs. C_D90 _= –1.2 ± 1.2 D, *P* = 0.6; Stand-alone: C_D0_ = –1.6 ± 1.3 D vs. C_D90_ = –1.6 ± 1.2 D, *P* = 0.9), and no relevant time–by–group interaction was observed. Multivariate astigmatism analysis (*M*, *J*_0_, *J*_45_) indicated a significant change in the phacoemulsification cohort (*P* = 0.0003), in contrast to the stand-alone procedure cohort (*P* = 0.5). In the phacoemulsification cohort, *B* significantly declined from 2.9 ± 2.8 D to 1.2 ± 1.0 D (*P* < 0.0001) but remained stable in the stand-alone group (2.7 ± 3.5 D to 2.8 ± 3.4 D, *P* = 0.07). Corneal astigmatism analysis, based on 55 eyes from 37 patients, showed that the magnitudes of the *J*_0_ and *J*_45_ vectors remained stable over time for both the anterior (D0: *J*_0 _= –0.12 ± 0.07 D, D90: *J*_0 _= –0.21 ± 0.07 D, *P* = 0.12; D0: *J*_45 _= –0.05 ± 0.05 D, D90: *J*_45 _= –0.01 ± 0.05 D, *P* = 0.5) and posterior surfaces (D0: *J*_0 _= 0.12 ± 0.01 D, D90: *J*_0 _= 0.13 ± 0.01 D, *P* = 0.3; D0: *J*_45_ = –0.01 ± 0.01 D, D90: *J*_45 _= –0.01 ± 0.01 D; *P* = 0.9), without significant effects of time, group, or their interaction.

**Conclusions:**

The Hydrus Microstent is neutral in refractive astigmatism when used as a stand-alone procedure. In contrast, combined implantation with phacoemulsification can induce changes in refractive astigmatism. Keratometry analysis confirmed that both anterior and posterior corneal astigmatism remain stable, indicating that refractive changes are likely due to internal factors associated with lens exchange rather than the stent itself. Therefore, when implanted alone, the Hydrus Microstent is unlikely to compromise the results of prior refractive treatments.

## Introduction

Despite considerable efforts, glaucoma remains one of the most common causes of irreversible blindness worldwide [1]. Intraocular pressure (IOP) is recognized as the most important modifiable risk factor in glaucoma, and glaucoma treatment relies primarily on medical and surgical strategies to reduce and stabilize IOP [2]. Microinvasive glaucoma surgery (MIGS) includes devices that are designed to shunt aqueous outflow into Schlemm’s canal, the suprachoroidal or subconjunctival space [3–5]. In primary open-angle glaucoma eyes, aqueous outflow obstruction presumably relies on resistance-increasing trabecular meshwork alteration [2,6]. The Hydrus Microstent (Alcon Inc., Geneva, Switzerland) is precisely designed for implantation into Schlemm’s canal, thus creating direct access to the canal and increasing the outflow facility of the trabecular meshwork in this quadrant [7,8]. Recent research has shown that the Hydrus Microstent is safe and can substantially reduce outflow resistance and stabilize IOP [9,10].

Of note, the nasal and inferior quadrants of the trabecular meshwork have the highest density of collector channels associated with areas of active outflow [11]. Therefore, the Hydrus Microstent is most commonly implanted in these quadrants, as it maximizes the number of collector channels accessed beyond Schlemm’s canal. Implantation can be performed in combination with cataract surgery [12,13] or as stand-alone procedure [14].

Previous histological and scanning electron microscopy studies in non-human primates displayed no evidence of inflammation, granulation, or fibrosis in the aqueous outflow system or in surrounding tissues [15].

Nevertheless, the close proximity to inner corneal layers could allow the Hydrus Microstent to alter the corneal keratometry, for example, through extracellular matrix reduction, focal corneal oedema, or mechanical irritation of the chamber angle architecture, and ultimately to induce refractive changes.

Furthermore, the Hydrus Microstent is composed of nitinol, a shape-memory material. After implantation, it returns to its original shape and curvature. Notably, the asymmetrical force exerted by the quadrant-specific implantation on the surrounding structures could create local micromechanical tensions and deformations, ultimately leading to irregular shifts of the corneal curvature. As a consequence, irregular astigmatism components, as well as axis deviations, are conceivable, which are trackable using enhanced methods such as three-dimensional (3D) power-vector analysis. The aim of this study was to investigate the impact of Hydrus Microstent implantation on the occurrence or development of postoperative refractive and corneal astigmatism using a vector analysis.

## Methods

### Study design

This retrospective, non-randomized, uncontrolled, observational cohort study aimed to assess the short-term refractive and corneal astigmatism in glaucoma or ocular hypertension patients receiving Hydrus Microstent implantation as a stand-alone procedure or combined with phacoemulsification. Ophthalmological examination was scheduled preoperatively at baseline or day 0 (D0) and 90 days postoperatively (D90) and included slit-lamp microscopy, dilated fundus examination, Goldmann applanation tonometry, subjective refraction (sphere, cylinder, and axis), and corrected-distance visual acuity (CDVA, in LogMAR units [logarithm of the minimum angle of resolution]). Keratometry was not a prerequisite for study inclusion and was only analysed when available directly preoperatively and at D90 (Pentacam, OCULUS Optikgeräte GmbH, Wetzlar, Germany).

Exclusion criteria included myopia of more than –6 diopters (D), hyperopia of more than +4 D, an axial length shorter than 22 and longer than 26 mm, a sulcus- or scleral-fixated IOL implantation, and eye surgery within 6 months or cataract surgery within 3 months prior to implantation of the Hydrus Microstent. Patients with a CDVA of 0.6 LogMAR or worse, as well as patients with serious general diseases, were also excluded.

Written informed consent was obtained. The study adhered to the tenets of the Declaration of Helsinki and was approved by the local Institutional Review Board (16/17, Ethikkommission bei der Ärztekammer des Saarlandes). For research purposes, data were accessed on December 15^th^, 2024, June 30^th^, 2025, and November 15^th^, 2025. The authors had access to information that could identify individual participants during or after data collection.

### Hydrus microstent

The Hydrus Microstent is designed to bypass the trabecular meshwork while dilating roughly 3 clock hours of Schlemm’s canal. Full details of the Hydrus Microstent have been reported previously [10]. The long-term biocompatibility of this nitinol-based aqueous drainage device has been demonstrated [15]. Its curvature matches that of Schlemm’s canal.

The surgeon introduces the device via the trabecular meshwork, whereby the inlet segment is placed within the anterior chamber. The remaining scaffolding segments are located within 3 clock hours or a quadrant of Schlemm’s canal, aiming to impact the draining area of several collector vessels [16].

In 2011, the Hydrus Microstent received Conformité Européenne approval as both a stand-alone procedure and combined with phacoemulsification/cataract surgery.

### Surgical technique

The Hydrus Microstent is intended for *ab interno* implantation through the trabecular meshwork into Schlemm’s canal, as described in detail by Samuelson *et al*. [10] and Pfeiffer *et al*. [13]. All Hydrus Microstent implantation procedures were carried out by four surgeons with substantial experience in this technique. Correct positioning, defined as full placement of the stent within the Schlemm’s canal except for the inlet aperture, was assessed intraoperatively using a Swan-Jacobs gonioprism.

For the stand-alone procedure, two 1.2 mm wide clear-cornea side-port incisions were created. When combined with phacoemulsification, an additional temporal 2.4 mm clear-cornea main incision was created. The SENSAR™ Monofocal 1-Piece IOL (Johnson & Johnson) was implanted in all patients undergoing the combined procedure.

### Power vector analysis

Refractive astigmatism was displayed using a 3D power-vector system. As recommended by Thibos and Horner [17], we used three vector components: 1^st^, *M*, the spherical equivalent; 2^nd^, *J*_0_, the Jackson cross-cylinder C projection to the 0° and 90° meridian; and 3^rd^, *J*_45_, the C projection to the 45° and 135° meridian. The following formulae were used for calculation, where S is the sphere (D), C is the cylinder (D), and α is the axis (°).


M=S+C/2

$$J_{\text{0}}=(-\;\text{C}/\text{2})\;\text{cos}\;(\text{2}\alpha )$$

$$J_{\text{45}}=(-\;\text{C}/\text{2})\;\text{sin}\;(\text{2}\alpha )$$

To determine the impact of surgery on astigmatism, we also calculated the blurring power vector, *B* [17]. *B*_change_ represents the change in *B* between two time points.


$$B=\sqrt{M^{\text{2}}+\;{J_{\text{0}}}^{2}+\;{J_{\text{45}}}^{2}}$$

$$B_{change}=\sqrt{{\left(M-M^{{}^{\prime }}\right)}^{\text{2}}+\;{{(J}_{\text{0}}-{\text{J}}_{0}^{{}^{\prime }})}^{2}+\;{{(J}_{\text{45}}-J_{45}^{{}^{\prime }})}^{2}}$$

Keratometry-derived corneal anterior and posterior surface astigmatism analysis was performed using the vectorial parameters *J*_0_, *J*_45_, and *B*, calculated as follows:


$$B=\sqrt{{J_{\text{0}}}^{2}+\;{J_{\text{45}}}^{2}}$$

### Statistical analysis

The Shapiro–Wilk test was used to assess normality of univariate datasets. Quantitative and qualitative baseline characteristics of the phacoemulsification and stand-alone procedure cohorts were compared using the Mann–Whitney *U* test and Fisher’s exact test, respectively. The Wilcoxon signed-rank test was used to detect time course in CDVA.

Multivariate power-vector analysis of refractive astigmatism involved the three power-vector components: *M*, *J*_0_, and *J*_45_. Henze-Zirkler’s and Royston’s methods were used to test normality for multivariate datasets. The nonparametric rank-sum test was used for time course analysis of the paired datasets.

Because both eyes from the same patient could be included, and repeated measurements were available over time (D0 and D90), we accounted for within-subject correlation using linear mixed-effects models (LMM) for both refractive and keratometry-derived corneal anterior and posterior surface astigmatism analysis. Patient identification number (ID) and surgeon identity were included as random effects, whereas time and/or surgical group was included as a fixed effect. Intraoperative complications were incorporated as an additional variable. Multivariate linear mixed-effects regression (MLR) was used to investigate the influence of age, sex, M_D0_, and IOP on *B*_Change_, with patient ID included as a random effect.

A post hoc power analysis was conducted using G*Power (version 3.1), applying the “Means – Wilcoxon signed-rank test (matched pairs)” module to assess the statistical power for detecting changes in *B* over time.

Data are reported as mean ± standard deviation for descriptive purposes. *P* values were two-sided and considered statistically significant when < 0.05. MATLAB was used for multivariate analysis (version R2023b, MathWorks, Inc). JMP (version 16.2.0, SAS Institute, Inc.) was used for the remaining statistical analyses and for generating double-angle plots.

## Results

### Subjects

The Hydrus Microstent was successfully implanted in 90 eyes from 74 patients. Thirty eyes underwent stand-alone implantation, while 60 eyes received the stent combined with cataract surgery. Table 1 summarizes the baseline characteristics for all eyes in both cohorts.

**Table 1:** Distribution of patient characteristics receiving the Hydrus Microstent in combination with phacoemulsification or undergoing implantation as a stand-alone procedure.

	Total Cohort	Phacoemulsification Cohort	Stand-alone Procedure Cohort	*P*-value
**Total**				
Eyes	90	60	30	
Patients	74	48	26	
**Sex**				
Male	34	23	11	0.5^#^
Female	40	25	15	
**Age** (years)	70.0 ± 10.3	68.7 ± 7.4	72.2 ± 14.1	0.03*
**Eye**				
Right	45	30	15	0.9^#^
Left	45	30	15	
**Intraocular Pressure** (mmHg)	19.7 ± 7.2	19.3 ± 5.8	20.7 ± 9.6	0.9*
**Diagnosis**				
Chronic Primary Open-Angle Glaucoma	47	31	16	0.06^#^
Normal Tension Glaucoma	7	6	1	
Pseudoexfoliative Glaucoma	17	11	6	
Pigmentary Glaucoma	6	5	1	
Uveitic Glaucoma	1	1	0	
Rubeotic Glaucoma	0	0	0	
Aphakia Glaucoma	0	0	0	
Juvenile Glaucoma	0	0	0	
Angle Closure Glaucoma	3	3	0	
Traumatic Glaucoma	1	0	1	
Ocular Hypertension	8	3	5	
**Previous Glaucoma Surgery**				
Trabeculectomy	1	0	1	0.6^#^
Trabeculotomy	1	1	0	
Canaloplasty	2	1	1	
**Lens Status**				
Phakia	69	60	9	< 0.0001^#^
Pseudophakia	21	0	21	
**Ocular Comorbidity**				
Amblyopia	2	1	1	0.1^#^
Exudative Age-related Macular	7	4	3	
Degeneration				
Dry Geographical Atrophy	1	1	0	
Diabetic Macular Oedema	0	0	0	
Irvine Gass Oedema	2	1	1	
Epiretinal Gliosis	2	2	0	
Vascular Occlusion	4	2	2	
Lamellar Macular Hole	4	4	0	
Full Thickness Macular Hole	1	1	0	
Anterior Ischemic Neuropathy	1	0	1	
Keratoconus	1	1	0	
**Intraoperative Complications**				
Intracameral Bleeding	19	15	4	<0.0001^#^
Multiple Implantation Attempts	8	2	6	
Device Repositioning	6	6	0	

Patients’ sex and age, laterality (right or left), intraocular pressure, diagnosis, previous glaucoma surgery, lens status, visual acuity reducing ocular comorbidities, and intraoperative complications were recorded. ^#^ Fisher’s exact test, * Mann–Whitney *U* tests comparing the phacoemulsification with the stand-alone procedure cohort.

The preoperative CDVA was 0.4 ± 0.4, 0.3 ± 0.5, and 0.4 ± 0.4 LogMAR for the total, stand-alone, and phacoemulsification groups, respectively. By postoperative day 90, CDVA improved to 0.2 ± 0.4 in both the total and phacoemulsification cohorts (*P* < 0.0001, Wilcoxon signed-rank test), while remaining unchanged in the stand-alone cohort (0.3 ± 0.5, *P* = 0.4, Wilcoxon signed-rank test).

No major complications occurred. Minor events were infrequent and mild, and are summarized in Table 1. Two cases of postoperative macular edema occurred in the phacoemulsification group.

### Spherical equivalent

The spherical equivalent M did not significantly change between baseline and 3 months. In the overall cohort, M_D0_ was –1.5 ± 3.8 D and M_D90_ was –1.1 ± 2.5 D (*P* = 0.3, LMM). Baseline values were similar between the phacoemulsification (M_D0_ = –1.2 ± 3.8 D) and stand-alone groups (M_D0_ = –2.0 ± 3.8 D, *P* = 0.1, LMM), and neither subcohort showed a significant change over time (Phacoemulsification: M_D90_ = –0.6 ± 1.2 D; Stand-alone: M_D90_ = –1.9 ± 3.8 D; both *P* > 0.2, LMM).

### Refractive astigmatism

In the total cohort, refractive cylinder did not change over time, measuring –1.4 ± 1.1 D at baseline and –1.3 ± 1.2 D at 3 months (*P* = 0.7, LMM). Baseline values were comparable between the phacoemulsification group (C_D0_ = –1.3 ± 0.9 D) and the stand-alone group (C_D0_ = –1.6 ± 1.3 D, *P* = 0.2, LMM). Neither group showed a significant change in refractive cylinder over time (Phacoemulsification: C_D90 _= –1.2 ± 1.2 D, *P* = 0.6, LMM; Stand-alone: C_D90_ = –1.6 ± 1.2 D, *P* = 0.9, LMM), and no relevant time–by–group interaction was observed.

**Fig 1:**
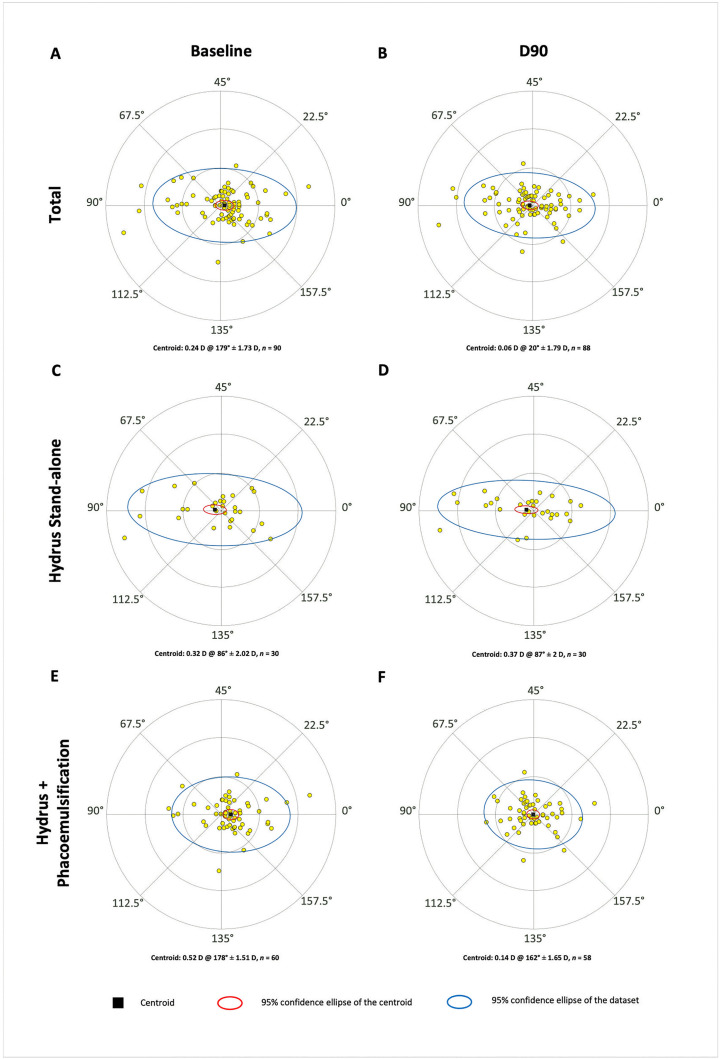
Double-angle plots illustrating refractive astigmatism at baseline (A, C, and E) and 90 days postoperatively (B, D, and F) for all eyes (A and B) and both subcohorts separately (*i.e*., stand-alone [C and D] and phacoemulsification [E and F]). Centroid values (written and graphically represented as black squares), standard deviations, and 95% confidence ellipses for both the centroid (in red) and dataset (in blue) are displayed.

Fig 1 displays double-angle plots showing refractive astigmatism at baseline and D90.

In the phacoemulsification cohort, multivariate power-vector analysis (*M*, *J*_0_, *J*_45_) demonstrated a significant shift in refractive astigmatism over time (*P* = 0.0003, rank-sum test), whereas no significant change was observed in the stand-alone procedure cohort (*P* = 0.5, rank-sum test).

Overall, the average magnitude of the vector *B* was 2.9 ± 3.0 D at baseline and decreased to 1.8 ± 2.3 D at D90 (*P* = 0.0003, LMM). In the phacoemulsification cohort, *B* significantly declined from 2.9 ± 2.8 D to 1.2 ± 1.0 D (*P* < 0.0001, LMM) but remained stable in the stand-alone group (2.7 ± 3.5 D to 2.8 ± 3.4 D, *P* = 0.07, LMM).

The average *B*_Change_ vector (*M* – *M*’, *J*_0_ – *J*_0_’, *J*_45_ – *J*_45_’) was 1.9 ± 2.0 D in the overall cohort and, consistently with the behavior of *B*, was significantly larger in the phacoemulsification group (*B*_Change_ = 2.6 ± 2.2 D) than in the stand-alone group (*B*_Change_ = 0.6 ± 0.4 D, *P* < 0.0001 LMM). The MLR identified only M_D0_ as a significant predictor of *B*_Change_, with a more myopic M_D0_ being associated with a larger reduction in *B* (R^2^ = 0.16, *P* < 0.0001), whereas age (R^2^ = 0.009, *P* = 0.4), sex (R^2^ < 0.001, *P* = 0.9), and baseline IOP (R^2^ < 0.0001, *P* = 0.9) showed no influence.

Regarding individual power-vector components, *J*_0_ showed small but significant changes over time and between groups. Overall, *J*_0_ increased slightly from –0.07 ± 0.6 D at D0 to 0.04 ± 0.6 D at D90 (*P* = 0.02, LMM) and was marginally more “with-the-rule” in the stand-alone group than in the phacoemulsification group (*P* = 0.04, LMM). However, the absolute shift magnitude was < 0.2 D and is thus unlikely to be clinically meaningful. No significant effects of time, group, or their interaction were detected for *J*_45_ (*P* > 0.5, LMM).

### Corneal astigmatism

A total of 55 eyes from 37 patients had preoperative and postoperative keratometry data. Twenty eyes from 18 patients, average age 74.1 ± 11.0 years, including 7 men and 11 women, underwent stand-alone implantation. Thirty-five eyes from 29 patients, average age 68.9 ± 7.2 years, including 14 men and 15 women, underwent implantation combined with phacoemulsification.

**Fig 2:**
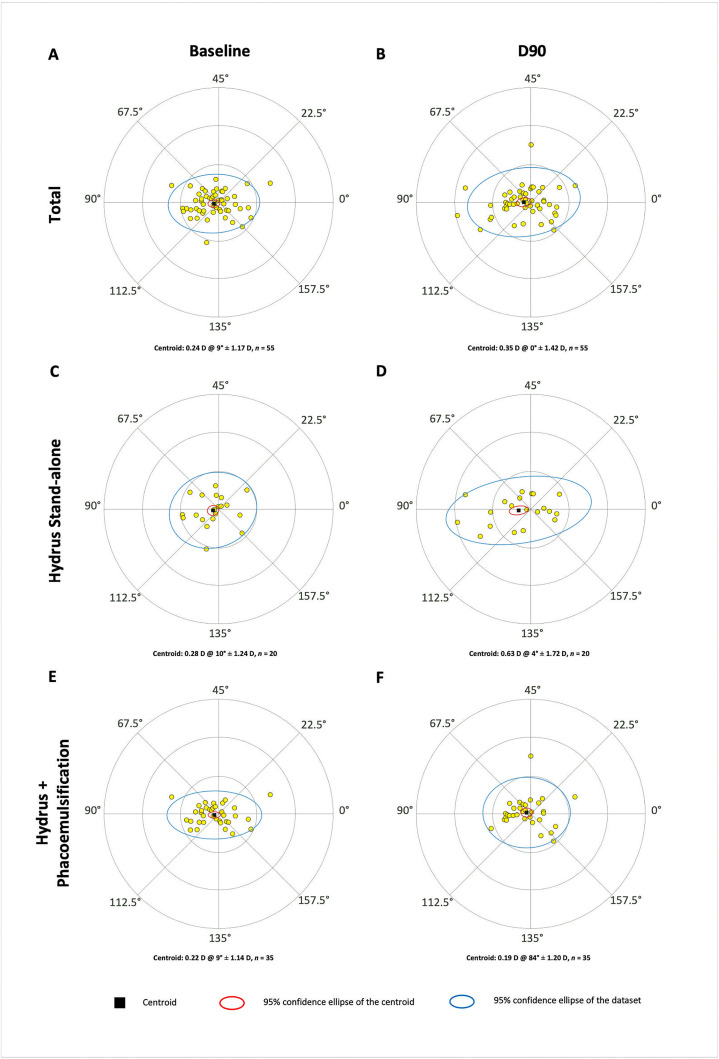
Double-angle plots illustrating anterior surface corneal astigmatism at baseline (A, C, and E) and 90 days postoperatively (B, D, and F) for all eyes (A and B) and both subcohorts separately (*i.e*., stand-alone [C and D] and phacoemulsification [E and F]). Centroid values (written and graphically represented as black squares), standard deviations, and 95% confidence ellipses for both the centroid (in red) and dataset (in blue) are displayed.

Anterior surface *J*_0_ remained stable over time in the overall cohort (D0: *J*_0 _= –0.12 ± 0.07 D; D90: *J*_0 _= –0.21 ± 0.07 D, *P* = 0.12, LMM), without significant effects of time, group, or their interaction. Anterior surface *J*_45_ was close to zero throughout the investigation (D0: *J*_45 _= –0.05 ± 0.05 D; D90: *J*_45 _= –0.01 ± 0.05 D, *P* = 0.5, LMM), without significant effects of time, group, or their interaction. The magnitude of anterior surface vector *B* was 1.02 ± 0.10 D at D0 and 1.25 ± 0.10 D at D90. This time course reached statistical significance (*P* = 0.007, LMM), but did not vary between groups at any time point. Fig 2 displays the respective double-angle plots of corneal anterior surface astigmatism.

Posterior surface *J*_0_ was stable near zero both at baseline (*J*_0 _= 0.12 ± 0.01 D) and at 3 months (*J*_0 _= 0.13 ± 0.01 D, *P* = 0.3, LMM), as was *J*_45_ (D0: *J*_45 _= –0.01 ± 0.01 D; D90: *J*_45 _= –0.01 ± 0.01 D; *P* = 0.9, LMM). The magnitude of posterior surface vector *B* was similarly unchanged (D0: *B* = 0.29 ± 0.2 D; D90: *B* = 0.32 ± 0.02 D, *P* = 0.3, LMM), without significant effects of time, group, or their interaction.

### Impact of intraoperative complications

There was no significant effect of complication type on any postoperative corneal or refractive astigmatism parameter (*P* > 0.4). Least-squares means for all power-vector components were similar across complication categories, with broadly overlapping confidence intervals.

### Post hoc power analysis

A post hoc power analysis based on the total sample size of 90 eyes, an alpha level of 0.05, and an estimated effect size (dz) of approximately 0.4 yielded a statistical power of 0.96 for detecting changes in *B* between the two time points.

## Discussion

The objective of this retrospective, non-randomized, uncontrolled, observational cohort study was to evaluate short-term postoperative astigmatism following stand-alone Hydrus Microstent implantation and in combination with phacoemulsification. Although prior studies have investigated the performance and safety of the Hydrus Microstent [9,10,13], detailed astigmatism analyses have not been conducted. However, this is particularly important because astigmatism changes resulting from Schlemm’s canal interventions can impact visual acuity and ultimately affect the patient satisfaction and quality of life.

Subconjunctival MIGS, such as XEN GelStent and Preserflo Microshunt, as well as ab interno trabecular procedures like microhook trabeculotomy, have been shown to have minimal impact on astigmatism [18,19]. Nonpenetrating glaucoma surgery, such as canaloplasty, can induce transient postoperative astigmatism that typically returns to baseline within six months [20,21], while even suprachoroidal telemetric devices for long-term IOP monitoring are astigmatism-neutral [20,22–24]. However, nitinol, the shape-memory material comprising the Hydrus Microstent, does not adapt to individual anterior segment anatomy, potentially affecting the curvature of the surrounding layers and ultimately inducing astigmatism.

Multivariate 3D power-vector analysis (*M*, *J*_0_, *J*_45_), which provides a robust assessment of astigmatic changes, showed no significant variation between baseline and D90 in the stand-alone cohort, with the *B*_Change_ vector averaging only 0.6 ± 0.4 D. In contrast, the phacoemulsification cohort demonstrated a significant time course, with a *B*_Change_ vector of 2.6 ± 2.2 D.

Surgically induced astigmatism is influenced by incision length, type, location, and particularly incision width [25,26]. The 2.4 mm main incision used in phacoemulsification may therefore have contributed to the observed refractive changes, whereas the 1.2 mm side-port incisions are considered astigmatism-neutral [27,28]. Clear corneal incision placement and subsequent wound healing can both influence postoperative astigmatism [29]. Notably, surgically induced astigmatism typically decreases within the first postoperative month, which supports the timeline chosen in this study [27,28]. Despite these considerations, keratometry analysis confirmed complete stability of both anterior and posterior corneal astigmatism components *J*_0_ and *J*_45_. The small, though significant, variations observed in the anterior surface vector *B* likely reflect the inherent variability of measuring low-magnitude corneal astigmatism, influenced by slight alterations in tear film, ring reflections, axis determination, or even minor decentrations, rather than structural changes. Although these keratometry-specific results are derived from a limited sample and warrant confirmation in larger prospective studies, they suggest that the Hydrus Microstent does not induce systematic corneal alterations, and that refractive changes after combined surgery are primarily attributable to internal optical factors related to lens extraction and IOL implantation, such as spherical power shifts or lens tilt.

Inclusion of the variable “intraoperative complications” in LMMs revealed no significant association with any refractive or keratometric parameters, indicating that mild intraoperative events such as intracameral bleeding, multiple implantation attempts, or device repositioning did not measurably affect refractive stability at three months postoperatively.

Notably, age, sex, and baseline IOP had no impact on refractive *B*_Change_. Baseline spherical equivalent, M_D0_, showed an association, but this was expected given its inclusion in the *B*_Change_ vector calculation. Therefore, no adjustment was made in the main analysis.

These findings are important, as they show that Hydrus Microstent implantation—an emerging MIGS procedure with good performance—is astigmatism-neutral in the short-term. However, the study did not assess potential very early, transient shifts occurring within days to weeks, as have been reported with canaloplasty [20,21], which represents a notable limitation. Additional limitations include the retrospective design, a limited sample size, inclusion of several surgeons, and lack of long-term follow-up. However, post hoc power analysis indicated a power greater than 0.9, confirming statistical robustness, and surgeon identity did not significantly influence outcomes. Nevertheless, long-term follow-up is warranted to evaluate a potential sustained or late impact of Hydrus Microstent implantation on corneal and refractive astigmatism.

In conclusion, the Hydrus Microstent is neutral in refractive astigmatism when used as a stand-alone procedure. In contrast, when combined with phacoemulsification, shifts in refractive astigmatism may occur. Keratometry confirmed stable anterior and posterior corneal astigmatism, indicating that these changes are most likely related to internal optical factors of lens exchange. Thus, when implanted alone, the Hydrus Microstent is unlikely to compromise the outcomes of previous refractive treatments.

## Supporting information

S1 Table Raw subjective refraction data at baseline and at 3 months (D90). (PDF)
