# Supplementary material for: Short-term refractive and corneal astigmatism after canal-based microinvasive glaucoma surgery
Source: PLoS One. 2026 Jan 8;21(1):e0340377. doi: 10.1371/journal.pone.0340377 (PMC12782394; doi:10.1371/journal.pone.0340377)
Supplement: S1 Table — (PDF) [file pone.0340377.s001.pdf]

		Baseline			D90	
	Sphere	Cylinder	Axis	Sphere	Cylinder	Axis
Phacoemulsification	-0,75	-0,25	0	-0,25	-0,5	179
Phacoemulsification	+3,0	-1,25	63	1,25	-1,75	68
Phacoemulsification	-2,25	-0,25	101	-2,5	-0,5	159
Phacoemulsification	+2,50	-0,75	76	0,75	-1	90
Phacoemulsification	-0,75	-0,75	125	-0,75	-0,25	11
Phacoemulsification	+0,75	-0,5	90	-0,25	-0,5	136
Phacoemulsification	+3,0	-2,25	124	1	-2,25	141
Phacoemulsification	2,5	-0,5	111	0,5	-1,75	19
Phacoemulsification	0,0	-1	95	1	-1	132
Phacoemulsification	-7,25	-1	122	0,25	-0,5	144
Phacoemulsification	+0,50	0	0	0	-0,5	12
Phacoemulsification	-1,5	-3,5	95	1,25	-3,25	95
Phacoemulsification	1,5	-0,5	148	0,5	-0,75	156
Phacoemulsification	1,25	-1,5	101	-0,25	-1,5	101
Phacoemulsification	-5,25	-2,75	82	-1,50	-1,5	91
Phacoemulsification	-0,50	-2,5	85	0,25	-1,5	70
Phacoemulsification	2	-0,75	42	0,75	-1,25	18
Phacoemulsification	0,5	-2,5	99	0,5	-2,5	87
Phacoemulsification	-9,50	-1,75	75	-2,50	-0,75	9
Phacoemulsification	-9,25	-1	91	-2,50	-1,25	26
Phacoemulsification	-3,75	-1,25	125	1,25	-1	148
Phacoemulsification	0,25	-0,75	58	0	-0,75	23
Phacoemulsification	0,5	-1,25	125	-0,25	-0,5	86
Phacoemulsification	-3,75	-3	44	1	-2,5	39
Phacoemulsification	-3,0	-2,5	162	-2,25	-2,25	168
Phacoemulsification	-0,75	-0,5	74	-1,75	-0,25	127
Phacoemulsification	-2,50	-1	128	0,5	-1,25	173
Phacoemulsification	0,75	-4,75	96	2,25	-8	77
Phacoemulsification	2,25	-0,25	75	0	-0,5	53
Phacoemulsification	3,5	-0,75	64	-0,50	-0,75	151
Phacoemulsification	3	-1	113	-0,25	-0,5	147
Phacoemulsification	-0,25	-2	92	2	-1,5	95
Phacoemulsification	0	-0,75	92	1	-0,25	69
Phacoemulsification	-9,2	-2,75	177	-2,25	-2	170
Phacoemulsification	-8,50	-2,5	1	-1,75	-2,5	5
Phacoemulsification	2,5	-0,5	84	-2,0	-1	155
Phacoemulsification	-1,75	-0,75	82	0,5	-1,25	6
Phacoemulsification	-1,0	-0,25	95	0,5	-0,5	176
Phacoemulsification	-10,25	-2,25	180	-1,50	-1,75	6
Phacoemulsification	0	-1	64	-1,00	-0,25	32
Phacoemulsification	-0,75	-1	67	0,25	-0,5	54

Phacoemulsification	0	-0,5	98	-0,25	-0,25	51
Phacoemulsification	3,25	-1,5	98	1	-1	106
Phacoemulsification	1,75	-1,25	77	-1,0	-1,25	86
Phacoemulsification	1,75	-0,5	50	0,25	-0,5	63
Phacoemulsification	-4,25	-1	12	0,75	-0,75	173
Phacoemulsification	3,25	-1,5	170	0	-0,5	51
Phacoemulsification	3,25	-1,75	72	0,5	-1	71
Phacoemulsification	3,5	-0,75	167	0,25		
Phacoemulsification	0,5	-1	19	0,5	-0,75	82
Phacoemulsification	-1,5	-2,25	61	-1,5	-2,5	65
Phacoemulsification	0,5	-0,5	74	-0,25	-0,75	106
Phacoemulsification	1,25	-1	68	-0,25	-0,5	39
Phacoemulsification	1,75	-0,75	132	0,5	-0,75	148
Phacoemulsification	2,25	-0,75	51	0	-0,75	14
Phacoemulsification	-0,75	-0,75	133	1,25	-1,25	148
Phacoemulsification	-1,5	-2,5	84	2	-0,5	88
Phacoemulsification	4,75	-0,75	117	-0,75	-0,75	126
Phacoemulsification	3	-1	82	0,75	-0,75	82
Phacoemulsification	-3,75	-1,25	72			
Stand-alone	+2,25	-2	108	2,25	-0,25	136
Stand-alone	+0,50	-0,75	131	0,75	-0,5	12
Stand-alone	-17	-2	157	-16,25	-2,75	169
Stand-alone	+2,50	-5,25	8	2	-5	6
Stand-alone	-0,25	-0,5	137	0	-1	125
Stand-alone	+1,75	-0,25	3	2	-0,25	49
Stand-alone	+1,50	-0,25	125	2	-0,5	126
Stand-alone	-0,50	-0,75	88	0	-1	84°
Stand-alone	-0,25	-0,25	74	0	-1	78
Stand-alone	-0,5	-1	116	-0,25	-1,25	109
Stand-alone	0	-2,75	166	0,25	-2	171
Stand-alone	-0,25	-2,25	5	-0,75	-2,25	175
Stand-alone	-0,50	-1	88	-0,5	-0,5	82
Stand-alone	1,5	-0,25	125	2	-0,5	151
Stand-alone	-0,75	-2	74	-0,75	-2	82
Stand-alone	-1,0	-1	59	-1,25	-1,25	85
Stand-alone	0,25	-1,75	179	-0,75	-2,5	177
Stand-alone	-2,0	-1	34	-2,75	-1,5	38
Stand-alone	-1,5	-0,75	96	-1,25	-0,75	88
Stand-alone	-7,0	-0,5	166	-6,75	-0,25	6
Stand-alone	-1,25	-0,5	126	-1,25	-0,5	139
Stand-alone	0,5	-2	105	1,75	-2,5	93
Stand-alone	0,5	-2,25	81	0,5	-2	82
Stand-alone	0	-0,75	70	1	-1,75	87

Stand-alone	-5,0	-4,25	173	-4,50	-4,25	174
Stand-alone	-5,00	-4,25	2	-4,25	-4	177
Stand-alone	-2,25	-0,75	66	-2,50	-1,75	31
Stand-alone	-4,5	-2	179	-5	-1,75	179
Stand-alone	1,25	-3	75	0,75	-2	97
Stand-alone	-0,75	-0,5	153	-0,25	-0,75	168
